# The Use of Targeted Therapy in Pediatric Acute Lymphoblastic Leukemia: Exploring Novel Approaches and Emerging Therapies

**DOI:** 10.1007/s11864-025-01376-8

**Published:** 2026-02-23

**Authors:** David J. Byrwa, Marissa Krieger, Lisa M. Niswander, Kara M. Kelly

**Affiliations:** 1 Department of Pediatrics, Roswell Park Comprehensive Cancer Center, Buffalo, NY 14263 USA; 2 Department of Pediatrics, University at Buffalo Jacobs School of Medicine and Biomedical Sciences, Buffalo, NY 14203 USA

**Keywords:** Pediatric, Acute lymphoblastic leukemia, Targeted therapy, Immunotherapy, Toxicity

## Abstract

With modern risk stratification of multi-agent cytotoxic chemotherapy protocols, we now cure the majority of patients with pediatric acute lymphoblastic leukemia (ALL). However, patients with high-risk features or relapsed disease continue to have suboptimal outcomes. Conventional chemotherapy agents may increase long term organ toxicities even in more favorable patients. Improved understanding of the molecular characteristics of pediatric ALL has led to the preclinical and clinical development of more targeted and less systemically toxic therapeutic options for patients with ALL. Agents such as blinatumomab, inotuzumab ozogamicin, tisagenlecleucel and several others have revolutionized the treatment of relapsed or refractory ALL by either directing therapies locally to leukemic cells or by targeting specific leukemogenic pathways. Here we present current evidence for efficacy and toxicity profiles for these targeted therapies in pediatric patients with ALL. Many of these strategies have been more comprehensively studied in the adult population and we will highlight ongoing and needed clinical trials in pediatrics. We need to overcome our historically delayed approach to introducing new therapeutic options in pediatrics, as adults often benefit from innovations for years before they are evaluated in children. More proactively incorporating these emerging treatments into frontline therapy for the most vulnerable, high-risk pediatric ALL populations could meaningfully reshape our treatment paradigm. Earlier collaboration for development of clinical trials across pediatric and adult ALL may facilitate more rapid access to promising agents. We anticipate that successful upfront integration of more targeted approaches will improve response rates, reduce reliance on highly toxic chemotherapies and ultimately increase the likelihood of duration of remission. We expect the next generation of clinical trials will credential more targeted therapies for use in frontline therapy with the goal of improved outcomes with minimized toxicity for all patients with pediatric ALL, not just those with more favorable disease characteristics.

## Introduction

Acute lymphoblastic leukemia (ALL) is the most common pediatric cancer and the leading cause of disease-related mortality in childhood [1, 2]. Over the past 50 years, 5-year survival for pediatric patients younger than 15 years of age with newly diagnosed ALL has increased from 60% to 90%; drastically, the 5-year survival rate for adolescents 15–19 years of age increased from 28% to 75% [1, 3]. Despite these improvements derived from increased efficacy of frontline therapy, outcomes remain suboptimal for certain subsets of patients including infants less than 12 months of age at diagnosis, patients with relapsed T-cell ALL, and pediatric and young adult patients with relapsed or refractory (R/R) disease. Only approximately 50% of children and adolescents with first relapse of ALL experience long term survival and outcomes are even worse with second or greater relapses [4].

The standard of care in ALL treatment includes traditional chemotherapy given in multiple phases of treatment such as induction, consolidation, and maintenance [5]. Conventional chemotherapies with activity against both B- and T-ALL include corticosteroids, vincristine, asparaginase, doxorubicin, methotrexate, cyclophosphamide, cytarabine and mercaptopurine [5]. Unfortunately, patients that do not achieve clearance of MRD after conventional chemotherapy are more likely to relapse. In high-risk patients with relapsed or refractory disease, consolidation with allogeneic hematopoietic stem cell transplantation (allo-HSCT) is a potentially curative option, but with significant risk of toxicities. With the advent of improved understanding of pediatric ALL biology and more accessible comprehensive molecular diagnostic testing, new precision medicine approaches are in development along the preclinical and clinical trial pipeline with very encouraging results.

## Review of Targeted Therapies

**Fig. 1:**
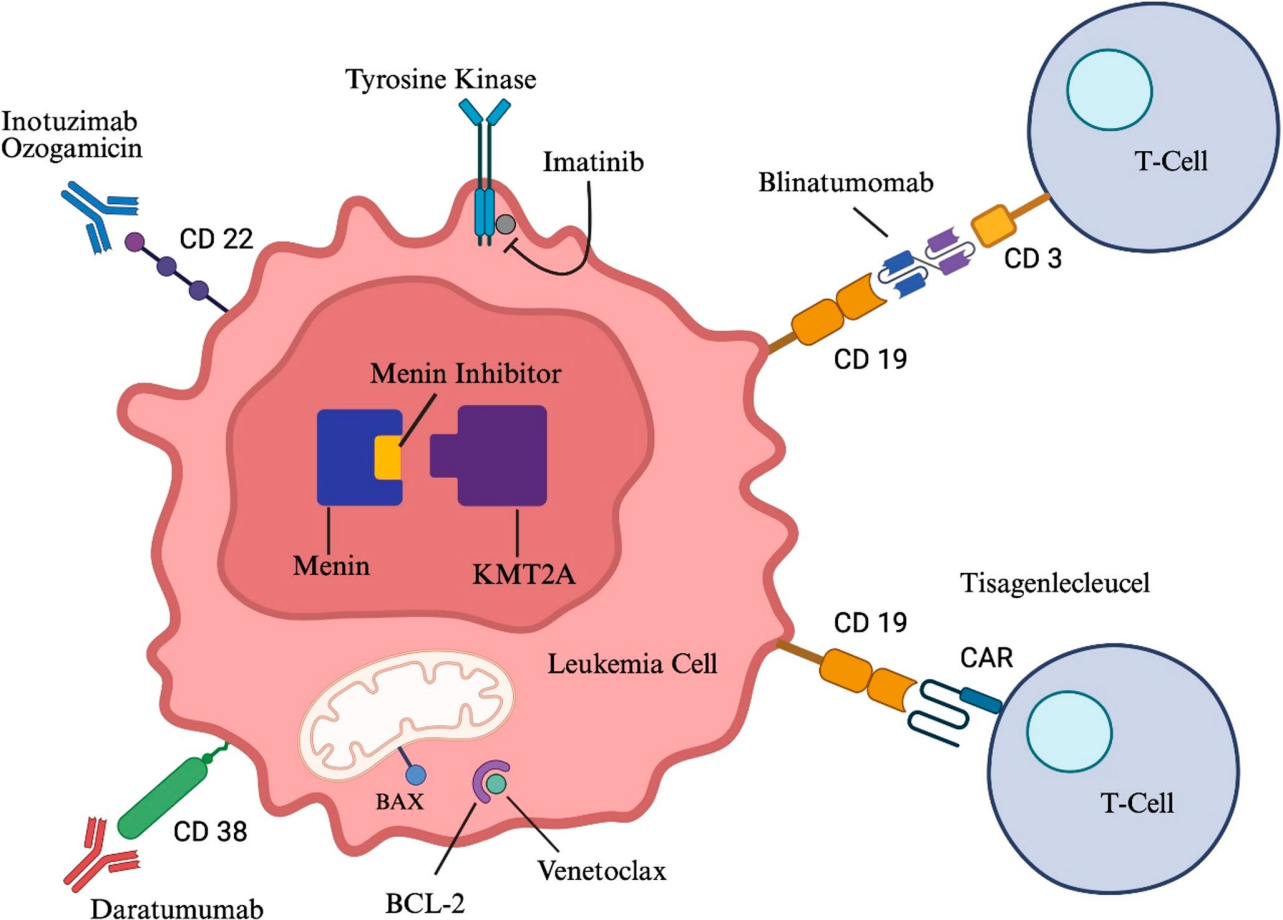
This image displays a leukemia cell and associated T cells with simple illustration of the mechanism of current targeted therapeutic options for pediatric acute lymphoblastic leukemia [13]

Targeted therapy refers to treatment approaches that are designed with specificity for a leukemia cell, whether via recognition of leukemia cell surface molecules or other intracellular alterations involved in growth and survival of leukemia cells. Figure 1 provides an illustration of the different mechanisms of targeted therapy. Immunotherapies like bispecific T-cell engagers (BiTE) and chimeric antigen receptor (CAR) T-cells direct a patient’s own immune system to identify and attack leukemia. Antibody drug conjugates like inotuzumab ozogamicin (INO) utilize antibody recognition of cell surface proteins to deliver toxic payloads directly to leukemia cells, and monoclonal antibodies like daratumumab target leukemia cells via multiple immune-mediated cytotoxicity mechanisms. Other small molecule inhibitors in development target activated tyrosine kinase signaling, pro-apoptotic pathways, or disrupt epigenetic regulation of leukemogenic gene expression. Given the specificity of these therapies for certain leukemia cell surface components or molecular alterations, molecular characterization to ensure appropriate patient selection is often necessary. Most of the therapies to be discussed are expected to achieve the best outcomes in combination with a traditional chemotherapy backbone. We will highlight critical mechanistic aspects of each targeted therapy, discuss ongoing and recent clinical trial results and FDA approvals in specific subsets of pediatric ALL patients, and highlight common or unique side effects for each modality. Table 1 provides a concise display of where each of these therapies can be used in treatment along with type of formulation and current recommended dosage.

**Table 1:** This table displays each discussed therapy type with corresponding medication name, current indication in therapy, formulation and recommended dosage [6–12]. * Indicates FDA approval for use in pediatric patients

Therapy Type	Examples	Indication	Formulation	Recommended Dosage
Anti-CD19/CD3 BiTE	Blinatumomab*	R/R B-ALL, SR B-ALL	IV infusion	< 45 kg: 15 mcg/m^2^/day, max 28 mcg/day > 45 kg: 28 mcg/day (Continuous for 28 days followed by a 14-day treatment-free interval)
Anti-CD22 Monoclonal Antibody	Inotuzumab ozogamicin*	R/R B-ALL, HR B-ALL	IV infusion	1.8 mg/m^2^ for cycle 1 (Day 1- 0.8 mg/m2, Day 8- 0.5 mg/m^2^, Day 15 − 0.5 mg/m^2^) Dosing for subsequent cycles dependent on remission status
CAR T-cell Immunotherapy	Tisagenlecleucel*	R/R B-ALL	IV infusion	≤ 50 kg: weight-based dose: 0.2–5.0 × 10^6^ CAR-positive viable T cells/kg > 50 kg: fixed dose: 0.1–2.5 × 10^8^ CAR-positive viable T cells
Tyrosine Kinase Inhibitor	Imatinib* Dasatinib* Ponatinib	Ph + ALL	Oral	Imatinib: 340 mg/m^2^/day, Max 600 mg/day Dasatinib: 10 – < 20 kg: 40 mg daily 20 – < 30 kg: 60 mg daily 30 – < 45 kg: 70 mg daily ≥ 45 kg: 100 mg daily
Menin Inhibitor	Revumenib*	KMT2Ar R/R ALL	Oral	< 40 kg: 160 mg/m^2^/dose ≥ 40 kg: 270 mg/dose Twice per day If patients are receiving strong CYP3A4 inhibitors: < 40 kg: 95 mg/m^2^/dose ≥ 40 kg: 160 mg/dose Twice per day
BCL-2 Inhibitor	Venetoclax	R/R ALL	Oral	No FDA approved dose
Anti-CD38 Monoclonal Antibody	Daratumumab	R/R T-cell ALL	IV infusion	No FDA approved dose

### Targeted Therapies with FDA Approval for Pediatric Patients

#### Blinatumomab, a Form of BiTE Therapy

Blinatumomab is a novel BiTE immunotherapy that binds to both CD19 on leukemia cells and to CD3 on T-cells, directing T-cell mediated cytotoxic death of the leukemia cells. Blinatumomab was approved in 2016 for pediatric patients with Philadelphia chromosome-negative relapsed or refractory B-ALL, and in 2024 as frontline consolidation in pediatric B-ALL patients over 1 month of age [3, 6]. There have now been several large cooperative group trials demonstrating improvement in outcomes for patients with childhood B-ALL in both the relapsed and upfront treatment settings.

Trial 20,120,215 was a global phase III trial across thirteen countries primarily in Europe (NCT02393859) which evaluated high-risk first relapse B-ALL patients in which pediatric patients were randomized to receive blinatumomab or conventional chemotherapy for the third consolidation cycle prior to HSCT. The study enrolled 108 pediatric patients ages 1–17 years old and the study was terminated early due to a prespecified stopping rule in favor of blinatumomab. The 5-year overall survival (OS) rate for the blinatumomab group was 78.4% compared to 41.4% in the chemotherapy only group; additionally, the 5-year relapse-free survival (RFS) rate for the blinatumomab group was 61.1% compared to 27.6% in the chemotherapy only group [14]. Similar results were observed with the Children’s Oncology Group (COG) trial AALL1331 which compared patients with low risk first relapse treated with chemotherapy alone versus chemotherapy plus blinatumomab (NCT02101853) [15]. 255 low risk patients after reinduction were included and patients randomized to the blinatumomab group received three 4-week blocks of blinatumomab. There was no statistically significant difference in disease free survival (DFS) or OS between the blinatumomab and standard chemotherapy arms overall, as patients with central nervous system (CNS) or testicular relapse fared poorly given limited CNS penetration of blinatumomab to these spaces [15]. However, there was a significant difference for patients with bone marrow ± extramedullary disease as blinatumomab significantly improved DFS and OS (72.7% compared to 53.7%) [15].

COG clinical trial AALL1731 (NCT03914625) studied the efficacy of the addition of blinatumomab to risk-adapted chemotherapy in newly diagnosed National Cancer Institute (NCI) standard-risk ALL patients. Patients were randomized to chemotherapy or chemotherapy plus two nonsequential cycles of blinatumomab. 1440 children were enrolled on the study. At a planned interim analysis, randomization was terminated early due to the surprising improvement in DFS. The estimated 3-year DFS rate for the chemotherapy only group was 87.9% while the chemotherapy and blinatumomab group had a DFS rate of 96% [3]. The transformative results of AALL1731 have led to the FDA approval for the incorporation of blinatumomab as standard of care for the majority of pediatric B-ALL patients.

An additional patient population who is expected to benefit from the addition of blinatumomab includes infants less than 12 months of age with B-ALL. The majority of infant ALL cases are characterized by a lysine (K)-specific methyltransferase 2 A (KMT2A) rearrangement, which is associated with aggressive disease and poor prognosis [16, 17]. An international, phase II study evaluated the addition of one post-induction, 28-day course of blinatumomab to the standard chemotherapy regimen, Interfant-06. The two-year disease-free survival for the blinatumomab group was 81.6%, compared to historical controls on the Interfant-06 trial with a 49.4% disease-free survival rate [16]. These promising results are currently being validated in a larger cohort in the phase III trial, Interfant-21 (NCT05327894).

Blinatumomab has a very favorable toxicity profile. A relatively unique side effect to immunotherapy that is generally seen within one week of starting blinatumomab is cytokine release syndrome (CRS), with rates of all grades ranging from 1.6 to 12% across the referenced trials [3, 6, 14, 15]. Due to the T-cell activation by blinatumomab, many inflammatory cytokines can be released and lead to downstream symptoms such as fever, headache, and rash [3]. In severe cases, hypotension, hypoxia, and organ dysfunction can also occur [18]. The severity of CRS with blinatumomab administration correlates to the amount of disease a patient has at time of administration. Patients with high tumor burden are more likely to have grades 3 and 4 CRS. CRS can be a life-threatening adverse effect and proper monitoring with early recognition of the signs and symptoms of CRS is a key point in its management.

In addition to CRS, blinatumomab can also have neurological toxicities, including immune effector cell-associated neurotoxicity syndrome (ICANS) [6]. ICANS typically occurs in the first week following initiation of therapy and it can present as a wide range of symptoms from headache or tremor to seizure or delirium. It is recommended that patients are monitored closely at initiation of blinatumomab and for the first week of infusion. The phase III trial 20,120,215 (NCT02393859) found overall incidence rates of neurologic events of 48.1% in the blinatumomab group compared to 29.4% in the consolidation chemotherapy group [14]. Management for neurotoxicity depends on the grade of the toxicity and therapy should be interrupted for any grade 3 or 4 toxicities [18]. Seizure prophylaxis is recommended in Down Syndrome patients receiving blinatumomab [19]. Clinical treatment protocols or institutional guidelines should be followed for management.

Standard risk children who received blinatumomab were more likely to experience grade 3 or greater sepsis and catheter-related infections than those who received standard chemotherapy alone [3]. Another notable adverse effect includes B-cell aplasia leading to hypogammaglobulinemia [6]. Use of immunoglobulin replacement should be based on clinical judgment.

Blinatumomab is administered as a continuous intravenous infusion over 28 days, a challenging administration schedule especially in young patients. While it is recommended that patients be monitored for the first week of therapy, most patients can continue the remainder of their treatment in the outpatient setting if resources are available [6]. The possibility of subcutaneous blinatumomab is under investigation and may help alleviate administration concerns in young patients [20].

#### Tisagenlecleucel, a CD19-targeting CAR T-cell Immunotherapy

CAR T-cell immunotherapy is an innovative approach where a patient’s own T cells are collected and then genetically engineered to express a CAR which recognizes B-cell/B-ALL surface protein CD19. Patients typically receive a bridging chemotherapy cycle aimed at achieving a low leukemia burden before starting lymphodepletion chemotherapy [21]. After the patient’s cell product is grown and lymphodepletion chemotherapy is completed, tisagenlecleucel is administered as a one-time intravenous infusion with recommended pre-medications [7]. The binding of CAR T-cells to ALL blasts results in T-cell activation and cytotoxic killing of leukemia cells [22].

In 2017, the FDA approved CD19-targeting autologous CAR T-cells tisagenlecleucel for treatment of patients up to 25 years of age with B-cell ALL that is refractory in second or later relapse and represented the first FDA approval in this novel class of therapies [7]. This approval was largely based on the ELIANA trial (NCT02435849), a single-cohort, phase II, global trial of tisagenlecleucel in children and young adults, aged 3–21 years old, with relapsed or refractory B-cell ALL [23]. This study reported an overall remission rate of 81% among 75 patients with heavily pre-treated relapsed or refractory disease, who had at least 3 months of follow-up after a single infusion of tisagenlecleucel [23]. At 3 year follow up, event free survival (EFS) and OS were 44% and 63%, respectively [24].

These findings coincided with a retrospective, multi-institutional study comprised of 185 pediatric and young adult patients with relapsed or refractory B-ALL. For those with high disease burden immediately prior to tisagenlecleucel therapy, 12-month OS was observed at 58% with EFS of 31% [25]. The data was more promising for those with lower disease burden prior to tisagenlecleucel, as those with low disease burden had OS of 85% and EFS of 70% [25]. These findings demonstrate favorable long-term efficacy and suggest tisagenlecleucel as an effective treatment in those with relapsed or refractory B-ALL with relatively low levels of disease. Tisagenlecleucel has not been prospectively studied to date in the infant ALL population, as children younger than 3 years of age were excluded from the ELIANA trial. Retrospective studies highlight that T-cell manufacture is feasible in the infant population and response rates appear comparable to older pediatric patients [26, 27]. Uniquely, patients with KMT2A-rearranged ALL have similar rates of relapse but are more likely to relapse with a leukemia lineage switch to acute myeloid leukemia (AML), which is associated with dismal outcomes [27, 28].

There are no absolute contraindications to use of tisagenlecleucel, though flow cytometry to ensure CD19 + disease is recommended. The most common nonhematologic adverse events reported were CRS and ICANS, both of which were described above [7]. In the ELIANA trial, CRS occurred in 77% of patients [23]. The patient is also at risk for hypersensitivity reaction, hypogammaglobulinemia, and prolonged cytopenia which can be associated with prolonged transfusion dependence and infectious risk [7]. There is also a warning about the risk of T-cell malignancy following treatment of a hematologic malignancy with CD19-directed immunotherapy, as a total of 22 adult cases of post infusion T-cell malignancies were described by the FDA, with malignant cells in 3 of the malignancies expressing the CAR transgene [29]. This has not been observed in the pediatric population.

#### Anti-CD22 Antibody Drug Conjugate

INO is an antibody-drug conjugate chemotherapy agent, originally approved in 2017 for adults with relapsed or refractory B-cell ALL. INO is a CD22-directed monoclonal antibody conjugated to cytotoxic antitumor antibiotic, calicheamicin [8]. By binding to CD22 on the leukemia cell surface, the complex is internalized and calicheamicin induces DNA double strand breaks, leading to apoptosis [8]. CD22 is also expressed on normal B-cells, which can also be targeted by INO.

With emerging therapies like INO in adults, an evaluation was conducted of pediatric patients who were treated with INO through the compassionate use program. Of the 51 patients who were treated, 67% had achieved a complete remission. INO was generally well tolerated, however grade 3 or greater adverse effects included liver dysfunction and infections. In this analysis, no cases of veno-occlusive disease (VOD) were observed due to INO, although of the 21 patients who proceeded to undergo an allo-HSCT, 11 patients were subsequently diagnosed with VOD [30].

COG study AALL1621 (NCT02981628) went on to examine the safety and efficacy of INO prospectively when used in the relapse or refractory setting for pediatric, CD22 positive B-ALL [31]. This was a single arm, phase II trial in which INO was administered to patients aged 1–21 years. Of the 48 evaluable patients on day 28, 19 achieved a complete response (CR) and 9 achieved a complete response with incomplete count recovery (CRi) [31]. The study concluded that INO was an effective treatment in previously treated patients. This study is ongoing with an additional cohort examining INO in combination with chemotherapy.

INO was FDA approved in March of 2024 for CD22-positive, relapsed or refractory B-cell ALL for pediatric patients 1 year of age or greater. Currently, the randomized phase III COG study AALL1732 (NCT03959085) is investigating the use of 2 blocks of INO intercalated in a standard chemotherapy backbone for patients with newly diagnosed high-risk B-cell ALL [31]. Planned interim analysis at two safety phases have led to modifications in dose and supportive care due to unacceptable rates of infection, treatment delays, and VOD [32]. Additional studies are recommended in infants after an international case study reported 7 of 15 patients under age 3 with relapsed or refractory B-ALL were able to achieve a complete remission [33].

Toxicity was significant in pediatric patients who received INO who then proceeded to HSCT, as grade III SOS/VOD was observed in 28.6% of these patients as part of AALL1621 [31]. Other common adverse events reported in AALL1621 included febrile neutropenia (29.2%) and infection (16.7%), with a smaller subset incurring transaminitis; all patients in this trial had peripheral B-cell aplasia [31].

#### Tyrosine Kinase Inhibitors (TKIs)

TKIs inhibit signal transduction pathways that mediate various cellular processes including growth and differentiation by blocking the action of specific enzymes known as tyrosine kinases [34]. In malignancies, aberrant tyrosine kinase activity can contribute to uncontrolled cellular proliferation. Imatinib, the first TKI approved for targeted cancer therapy, was initially studied in chronic myelogenous leukemia, a myeloproliferative cancer that is characterized by the Philadelphia chromosome [t(19;22)] and driven by BCR-ABL1 fusion [35]. There is a molecular subset of B-ALL harboring the Philadelphia chromosome (Ph + ALL), which confers poor prognosis in pediatric patients [36]. In addition, Philadelphia-like (Ph-like) B-ALL is a high-risk subset of pediatric B-ALL with a variety of genomic alterations that activate kinase or cytokine receptor signaling and have a gene expression profile that overlaps with Ph + ALL [37]. BCR-ABL1-targeting TKIs are administered as a daily oral agent in combination with chemotherapy in pediatric patients with confirmed Ph + ALL, in both newly diagnosed and relapsed or refractory disease [9]. Ongoing studies are evaluating TKIs in Ph-like ALL patients.

There was early promise with the use of TKIs in pediatric Ph + ALL, as COG AALL0031 showed 5-year disease-free survival of 70% for children and adolescents with Ph + ALL treated with first generation TKI imatinib plus intensive chemotherapy, with no advantage for allo-HSCT [38]. Results are forthcoming from EsPhALL2017/COG AALL1631 (NCT03007147), which compares the addition of imatinib to two different chemotherapy backbones in pediatric Ph + ALL.

Due to concern for acquisition of resistance mutations with imatinib, second generation (i.e. dasatinib) and third generation (i.e. ponatinib) TKIs have been created and studied. COG AALL0622 studied dasatinib plus chemotherapy in a cohort of 60 pediatric patients, with observed 5-year OS of 88%, which was similar outcome seen in AALL0031 [39]. The use of ponatinib has limited retrospective support in pediatrics but has been studied and FDA approved in adults with Ph + ALL. In a retrospective analysis of 12 pediatric patients with Ph + and Ph-like B-ALL treated with ponatinib alongside multiagent chemotherapy, 8 patients had an improvement in disease burden, one patient had stable disease, and three had refractory disease [40]. In another retrospective survey in Japan, 9 patients with Ph + ALL aged 8–16 years old received ponatinib, primarily as bridging therapy for HSCT, for varying lengths of time with other concomitant therapy. The response to ponatinib therapy was defined as achieving remission or a 2-log reduction in BCR-ABL1 mRNA level in patients already in remission; this occurred in 6 out of 9 patients [41]. Unfortunately, COG AALL1922, which looked at the use of ponatinib with chemotherapy with children, teenagers and adults with Ph + ALL, was terminated early due to dose-limiting liver toxicities [42]. Overall, there is no prospective data to comment on the effectiveness of ponatinib in pediatric Ph + B-ALL.

As it relates to TKI safety, the most frequently reported adverse reactions with imatinib include edema, cytopenia, nausea/vomiting, muscle cramps, rash and abdominal pain, according to package insert [10]. Similar side effects are seen in dasatinib as well [9]. As part of AALL0622 which studied dasatinib, 55% of patients had grade 3–4 infections [39]. Pleural effusions, a common side effect of dasatinib in the adult population, occurred in 9% of subjects on AALL0031 who received imatinib but was not seen in association with dasatinib on AALL0622. Neither imatinib nor dasatinib was associated with heart failure in pediatric patients [39].

#### Menin Inhibition

Menin inhibitors are a class of drugs that target binding of the adapter protein menin to KMT2A, which is a common driver in oncogenic fusions in KMT2A-rearranged infant ALL. The KMT2A-fusion-menin complex drives a pro-leukemogenic transcriptional program which is disrupted by menin inhibitors, leading to blast differentiation and apoptosis. Preclinical and early clinical studies have established the efficacy of this class of drugs against KMT2A-rearranged ALL and AML in adults and children [43]. Within the pediatric population there is much interest in clinical development of menin inhibitors for KMT2A-rearranged B-ALL, and in particular, infant ALL.

Revumenib is an oral medication and is the first FDA approved menin inhibitor and approval includes pediatric patients 1 year and older with relapsed or refractory KMT2A-rearranged acute leukemias. This approval was based on AUGMENT-101 (NCT04065399) which was a phase I/II, open-label, dose-escalation and expansion study of revumenib in adult and pediatric patients with heavily pretreated R/R KMT2A-rearranged acute leukemia [44]. In 23 children with ALL or AML who received revumenib monotherapy for a median duration of 10 weeks, 10 patients demonstrated a positive response (1 CR, 2 CRh (complete remission with partial hematologic recovery), 2 CR with incomplete platelet recovery, and 5 morphologic leukemia-free state) [44]. These are very promising results in this high-risk subgroup of pediatric leukemia patients who otherwise have a dismal prognosis. Ongoing COG study AALL2121 (NCT05761171) is a phase II trial of revumenib in combination with chemotherapy in relapsed or refractory KMT2A-rearranged leukemias and includes children 1 month – 6 years of age. Another menin inhibitor ziftomenib, which was designated with FDA breakthrough status in 2024, has demonstrated positive results in adults with AML, and is currently under phase I study in combination with chemotherapy in children with relapsed or refractory acute leukemia (NCT06376162) [45, 46].

AUGMENT-101 assessed for toxicity and found that 44.7% of patients experienced nausea, followed by febrile neutropenia (38.3%), diarrhea (35.1%), and QTc prolongation (25.5%) [11, 44]. QTc prolongation can be followed with periodic EKGs, which is recommended [11]. Of note, since revumenib is a substrate of cytochrome P450 3A4 (CYP3A4), the dose is adjusted if the patient is receiving a strong CYP3A4 inhibitor, such as certain antifungals [44].

### Selected Targeted Therapies Without FDA Approval for Pediatric Patients

#### BCL-2 Inhibition

Venetoclax is a BCL-2 inhibitor administered orally which works by displacing BH3-expressing pro-apoptotic proteins from BCL-2, thereby triggering apoptosis [47]. BCL-2 is overexpressed in B-ALL, providing rationale for BCL-2 inhibitor use. Venetoclax has been FDA approved for use in adults with chronic lymphocytic leukemia and AML [47]. The effectiveness of BCL-2 inhibitors such as venetoclax has not been established through clinical trials in pediatric patients with ALL, although its use in high-risk B-ALL and relapsed disease has been studied.

There is promising preclinical data and limited support obtained through retrospective chart review and a phase I clinical trial. Through in vivo study, the addition of venetoclax to conventional chemotherapeutic agents has been shown to result in survival advantage which illustrates potential to improve outcomes [48]. In a group of 18 pediatric and young adult patients with T-ALL, T-LBL or B-ALL who received venetoclax as part of combination therapy, 61% responded with CR/CRi [49]. Of these patients who responded, five had a diagnosis of T-cell ALL, five with T-cell lymphoblastic lymphoma, and 1 with B-ALL [49]. This case series demonstrates that venetoclax can be considered as a salvage chemotherapy option in pediatric patients with R/R ALL, particularly T-cell.

A recently published phase I, multicenter study (NCT03236857) in 31 pediatric and young adult patients less than 25 years evaluated venetoclax combined in chemotherapy with R/R ALL. The overall response rate of patients achieving a CR/CRi was 42%, with no response in four patients with T-ALL [50]. These patients were heavily pre-treated. Further studies using venetoclax in upfront therapy need to be performed.

In the retrospective study referenced above, the most common toxicity associated with venetoclax was thrombocytopenia, as 89% of patients developed grade 4 thrombocytopenia; other safety concerns were related to grade 4 neutropenia and grade 3 hyperbilirubinemia [12, 49]. Other studies have shown the most common adverse effects to be febrile neutropenia and sepsis [50, 51].

#### Anti-CD38 Monoclonal Antibody

CD38 protein is a transmembrane glycoprotein expressed on multiple immune cell types and plays a crucial role in regulating immune function [52]. Anti-CD38 monoclonal antibodies, such as daratumumab, work by inducing direct cytotoxic effects and enhancing immune-mediated destruction [53]. Daratumumab is given as an intravenous infusion with suggested use of pre-medications in the form of corticosteroids, antipyretics and antihistamines [54].

There is limited efficacy for use of daratumumab in pediatric malignancy. The DELPHINUS study was a phase II study evaluating the use of daratumumab in combination with chemotherapy in a cohort of patients aged 1–30 with R/R ALL with a primary endpoint of complete response [55]. 7 patients with B-ALL received treatment and no patient achieved CR while for the childhood T-cell ALL and young adult T-cell ALL cohorts, the CR rates at end of cycle one was 41.7% and 60.0%, respectively [55]. For a cohort of patients with T-ALL, daratumumab was reasonably effective for bridging to HSCT. More study is warranted.

In the DELPHINUS trial, the most common grade 3 or 4 adverse events across cohorts were hematologic events, such as anemia, leukopenia, and thrombocytopenia [55]. Febrile neutropenia occurred in 54% of patients, across all cohorts [55]. No new safety observations were made in the DELPHINUS study when compared with the adult population.

## Conclusion

The recent advances in targeted therapies for pediatric ALL have significantly expanded the treatment options available for children, particularly those with high-risk or relapsed disease. Challenges remain, including the potential for resistance, immune-related adverse events, and long-term toxicities. We have personally experienced the integration of these targeted therapies into clinical practice and this is a major step forward in the management of pediatric ALL. Continued research and clinical trials are essential to identify optimal treatment regimens and inform if targeted therapy can be utilized sooner in the upfront setting in patients with expected poor prognosis, not solely in the relapsed setting. The future of pediatric leukemia treatment lies in personalized, targeted approaches that combine the strengths of both traditional and novel therapies to achieve the best possible outcomes for young patients.

## Key References

- Gupta S, Rau RE, Kairalla JA, Rabin KR, Wang C, Angiolillo AL, et al. Blinatumomab in Standard-Risk B-Cell Acute Lymphoblastic Leukemia in Children. N Engl J Med. 2025;392(9):875-91.
  - ○ This phase III trial is notable because it demonstrated improved efficacy with use of blinatumomab as part of a standard risk regimen in pediatric patients with B-ALL rather than solely being used in the relapsed and refractory setting. The 3-year disease-free survival rate of the blinatumomab group was phenomenal as it reached 96%. This has revolutionized frontline therapy for this patient population.
- Laetsch TW, Maude SL, Rives S, Hiramatsu H, Bittencourt H, Bader P, et al. Three-Year Update of Tisagenlecleucel in Pediatric and Young Adult Patients With Relapsed/Refractory Acute Lymphoblastic Leukemia in the ELIANA Trial. J Clin Oncol. 2023;41(9):1664-9.
  - ○ The phase II ELIANA trial illustrated that tisagenlecleucel can be a highly efficacious and durable therapy in pediatric and young adult patients with relapsed or refractory B-ALL. Event free survival was 44% and overall survival was 63% at 3-year follow-up. It showed that immunotherapy could outperform chemotherapy or bone marrow transplant in certain settings.
- Issa GC, Aldoss I, Thirman MJ, DiPersio J, Arellano M, Blachly JS, et al. Menin Inhibition With Revumenib for KMT2A-Rearranged Relapsed or Refractory Acute Leukemia (AUGMENT-101). J Clin Oncol. 2025;43(1):75-84.
  - ○ The phase I/II AUGMENT-101 trial was the first trial to test menin inhibition in pediatric patients with R/R KMT2A-rearranged acute leukemia and it was associated with high remission rates, offering promise for a subset of pediatric leukemia which is classically difficult to cure with chemotherapy alone.

## Data Availability

No datasets were generated or analyzed during the current study.

## References

[1] Cancer Statistics R. 1975–2013 - Previous Version - SEER Cancer Statistics Review [Internet]. SEER. Available from: https://seer.cancer.gov/archive/csr/1975_2013/index.html

[2] Liu H, Xi R, Mao D, Zhao X, Wu T. Efficacy and Safety of Blinatumomab for the Treatment of Relapsed/Refractory Acute Lymphoblastic Leukemia: A Systemic Review and Meta-Analysis. Clin Lymphoma Myeloma Leuk. 2023;23(3):e139-e49. 10.1016/j.clml.2022.12.009 36593170

[3] Gupta S, Rau RE, Kairalla JA, Rabin KR, Wang C, Angiolillo AL et al. Blinatumomab in Standard-Risk B-Cell Acute Lymphoblastic Leukemia in Children. N Engl J Med. 2025;392(9):875 – 91. 10.1056/NEJMoa2411680 PMC11864901 39651791

[4] Rheingold SR, Bhojwani D, Ji L, Xu X, Devidas M, Kairalla JA, et al. Determinants of survival after first relapse of acute lymphoblastic leukemia: a Children’s Oncology Group study. Leukemia. 2024;38(11):2382–94. 39261601 10.1038/s41375-024-02395-4 PMC11518984

[5] Inaba H, Teachey D, Annesley C, Batra S, Beck J, Colace S, et al. Pediatric acute lymphoblastic leukemia, version 2.2025, NCCN clinical practice guidelines in oncology. J Natl Compr Canc Netw. 2025;23(2):41–62. 39938467 10.6004/jnccn.2025.0006

[6] Blincyto [package insert]. Thousand Oaks, California: Amgen: 2024.

[7] Kymriah [package insert]. East Hanover. New Jersey: Novartis Pharmaceuticals Corporation; 2025.

[8] Besponsa [package insert]. Philadelphia, PA: Wyeth Pharmaceuticals LLC: 2024.

[9] Sprycel [package insert]. Princeton, New Jersey: Bristol-Myers Squibb Company; 2024.

[10] Gleevec [package insert]. East Hanover. New Jersey: Novartis Pharmceuticals Corporation; 2022.

[11] Revuforj [package insert]. Waltham, MA: Syndax Pharmaceuticals, Inc; 2024.

[12] Venclexta [package insert]. North Chicago, IL: AbbVie Inc; 2024.

[13] Created in BioRender, Niswander L. (2025) https://BioRender.com/92361hf

[14] Locatelli F, Zugmaier G, Rizzari C, Morris JD, Gruhn B, Klingebiel T, et al. Effect of blinatumomab vs chemotherapy on Event-Free survival among children with High-risk First-Relapse B-Cell acute lymphoblastic leukemia: A randomized clinical trial. JAMA. 2021;325(9):843–54. 33651091 10.1001/jama.2021.0987 PMC7926287

[15] Hogan LE, Brown PA, Ji L, Xu X, Devidas M, Bhatla T, et al. Children’s oncology group AALL1331: phase III trial of blinatumomab in children, adolescents, and young adults with low-risk B-cell ALL in first relapse. J Clin Oncol. 2023;41(25):4118–29. 37257143 10.1200/JCO.22.02200 PMC10852366

[16] van der Sluis IM, de Lorenzo P, Kotecha RS, Attarbaschi A, Escherich G, Nysom K, et al. Blinatumomab added to chemotherapy in infant lymphoblastic leukemia. N Engl J Med. 2023;388(17):1572–81. 37099340 10.1056/NEJMoa2214171

[17] Clesham K, Rao V, Bartram J, Ancliff P, Ghorashian S, O’Connor D, et al. Blinatumomab for infant acute lymphoblastic leukemia. Blood. 2020;135(17):1501–4. 32043146 10.1182/blood.2019004008

[18] Jain T, Litzow MR. Management of toxicities associated with novel immunotherapy agents in acute lymphoblastic leukemia. Ther Adv Hematol. 2020;11:2040620719899897. 32010436 10.1177/2040620719899897 PMC6971963

[19] Li AM, Rabin KR, Kairalla J, Wang C, Devidas M, Militano O, et al. Blinatumomab associated seizure risk in patients with Down syndrome and B-lymphoblastic leukemia: an interim report from Children’s Oncology Group (COG) study AALL1731. Blood. 2021;138(Supplement 1):2304.

[20] Martínez Sánchez P, Zugmaier G, Gordon P, Jabbour E, Rifón Roca JJ, Schwartz S, et al. Safety and pharmacokinetics of subcutaneous blinatumomab (SC blinatumomab) for the treatment of adults with relapsed or refractory B cell precursor acute lymphoblastic leukemia (R/R B-ALL); results from a phase 1b study. Blood. 2022;140(Supplement 1):6122–4.

[21] Feuchtinger T, Bader P, Subklewe M, Breidenbach M, Willier S, Metzler M, et al. Approaches for bridging therapy prior to chimeric antigen receptor T cells for relapsed/refractory acute lymphoblastic B-lineage leukemia in children and young adults. Haematologica. 2024;109(12):3892–903. 38356450 10.3324/haematol.2023.283780 PMC11609793

[22] Awasthi R, Maier HJ, Zhang J, Lim S. Kymriah(R) (tisagenlecleucel) - an overview of the clinical development journey of the first approved CAR-T therapy. Hum Vaccin Immunother. 2023;19(1):2210046. 37185251 10.1080/21645515.2023.2210046 PMC10294746

[23] Maude SL, Laetsch TW, Buechner J, Rives S, Boyer M, Bittencourt H Tisagenlecleucel in Children and Young Adults with B-Cell Lymphoblastic Leukemia. N Engl J Med. 2018;378(5):439-48. 10.1056/NEJMoa1709866 PMC5996391 29385370

[24] Laetsch TW, Maude SL, Rives S, Hiramatsu H, Bittencourt H, Bader P et al. Three-Year Update of Tisagenlecleucel in Pediatric and Young Adult Patients With Relapsed/Refractory Acute Lymphoblastic Leukemia in the ELIANA Trial. J Clin Oncol. 2023;41(9):1664-9. 10.1200/JCO.22.00642 PMC10022844 36399695

[25] Bader P, Rossig C, Hutter M, Ayuk FA, Baldus CD, Bucklein VL, et al. CD19 CAR T cells are an effective therapy for posttransplant relapse in patients with B-lineage ALL: real-world data from Germany. Blood Adv. 2023;7(11):2436–48. 36607834 10.1182/bloodadvances.2022008981 PMC10242498

[26] Schultz LM, Baggott C, Prabhu S, Pacenta HL, Phillips CL, Rossoff J, et al. Disease burden affects outcomes in pediatric and young adult B-cell lymphoblastic leukemia after commercial Tisagenlecleucel: a pediatric real-world chimeric antigen receptor consortium report. J Clin Oncol. 2022;40(9):945–55. 34882493 10.1200/JCO.20.03585 PMC9384925

[27] Leahy AB, Devine KJ, Li Y, Liu H, Myers R, DiNofia A, et al. Impact of high-risk cytogenetics on outcomes for children and young adults receiving CD19-directed CAR T-cell therapy. Blood. 2022;139(14):2173–85. 34871373 10.1182/blood.2021012727 PMC8990372

[28] Silbert SK, Rankin AW, Hoang CN, Semchenkova A, Myers RM, Zerkalenkova EP, et al. Project EVOLVE: an international analysis of postimmunotherapy lineage switch, an emergent form of relapse in leukemia. Blood. 2025. 10.1182/blood.2024026655. 40193715 10.1182/blood.2024026655 PMC12333221

[29] Lamble AJ, Schultz LM, Nguyen K, Hsieh EM, McNerney K, Rouce RH, et al. Risk of T-cell malignancy after CAR T-cell therapy in children, adolescents, and young adults. Blood Adv. 2024;8(13):3544–8. 38701425 10.1182/bloodadvances.2024013243 PMC11261075

[30] Bhojwani D, Sposto R, Shah NN, Rodriguez V, Yuan C, Stetler-Stevenson M, et al. Inotuzumab Ozogamicin in pediatric patients with relapsed/refractory acute lymphoblastic leukemia. Leukemia. 2019;33(4):884–92. 30267011 10.1038/s41375-018-0265-z PMC6438769

[31] O’Brien MM, Ji L, Shah NN, Rheingold SR, Bhojwani D, Yuan CM, et al. Phase II trial of inotuzumab ozogamicin in children and adolescents with relapsed or refractory B-cell acute lymphoblastic leukemia: Children’s Oncology Group Protocol AALL1621. J Clin Oncol. 2022;40(9):956–67. 35007127 10.1200/JCO.21.01693 PMC8937013

[32] McNeer JL, O’Brien MM, Rheingold SR, Meenakshi Devidas, Chen Z, Bhojwani D, et al. A phase 3 randomized trial of inotuzumab Ozogamicin for newly diagnosed High-Risk B-ALL: safety phase results from children’s oncology group protocol AALL1732. Blood. 2021;138(Supplement 1):3398–8.

[33] Brivio E, Chantrain CF, Gruber TA, Thano A, Rialland F, Contet A, et al. Inotuzumab ozogamicin in infants and young children with relapsed or refractory acute lymphoblastic leukaemia: a case series. Br J Haematol. 2021;193(6):1172–7. 33529389 10.1111/bjh.17333

[34] Thomson RJ, Moshirfar M, Ronquillo Y. Tyrosine Kinase Inhibitors. StatPearls. Treasure Island (FL)2025. 33090752

[35] Cohen MH, Williams G, Johnson JR, Duan J, Gobburu J, Rahman A, et al. Approval summary for Imatinib mesylate capsules in the treatment of chronic myelogenous leukemia. Clin Cancer Res. 2002;8(5):935–42. 12006504

[36] Lee JW, Cho B. Prognostic factors and treatment of pediatric acute lymphoblastic leukemia. Korean J Pediatr. 2017;60(5):129–37. 28592975 10.3345/kjp.2017.60.5.129 PMC5461276

[37] Tran TH, Tasian SK. How i treat Philadelphia chromosome-like acute lymphoblastic leukemia in children, adolescents, and young adults. Blood. 2025;145(1):20–34. 38657263 10.1182/blood.2023023153 PMC12782968

[38] Schultz KR, Carroll A, Heerema NA, Bowman WP, Aledo A, Slayton WB, et al. Long-term follow-up of imatinib in pediatric Philadelphia chromosome-positive acute lymphoblastic leukemia: Children’s Oncology Group Study AALL0031. Leukemia. 2014;28(7):1467–71. 24441288 10.1038/leu.2014.30 PMC4282929

[39] Slayton WB, Schultz KR, Kairalla JA, Devidas M, Mi X, Pulsipher MA, et al. Dasatinib plus intensive chemotherapy in children, adolescents, and young adults with Philadelphia chromosome-positive acute lymphoblastic leukemia: results of Children’s Oncology Group Trial AALL0622. J Clin Oncol. 2018;36(22):2306–14. 29812996 10.1200/JCO.2017.76.7228 PMC6067800

[40] Rossoff J, Huynh V, Rau RE, Macy ME, Sulis ML, Schultz KR, et al. Experience with ponatinib in paediatric patients with leukaemia. Br J Haematol. 2020;189(2):363–8. 31975387 10.1111/bjh.16338

[41] Kodama Y, Sato A, Kato K, Sakaguchi H, Kato M, Kawasaki H, et al. Ponatinib in pediatric patients with Philadelphia chromosome-positive leukemia: a retrospective survey of the Japan Children’s Cancer Group. Int J Hematol. 2022;116(1):131–8. 35349077 10.1007/s12185-022-03329-5

[42] Clinicaltrials.gov. 2025 [cited 2025 Apr 21]. Available from: https://clinicaltrials.gov/study/NCT04501614

[43] Cuglievan B, Kantarjian H, Rubnitz JE, Cooper TM, Zwaan CM, Pollard JA, et al. Menin inhibitors in pediatric acute leukemia: a comprehensive review and recommendations to accelerate progress in collaboration with adult leukemia and the international community. Leukemia. 2024;38(10):2073-84. 10.1038/s41375-024-02368-7 PMC11436367 39179671

[44] Issa GC, Aldoss I, Thirman MJ, DiPersio J, Arellano M, Blachly JS, et al. Menin Inhibition With Revumenib for KMT2A-Rearranged Relapsed or Refractory Acute Leukemia (AUGMENT-101). J Clin Oncol. 2025;43(1):75-84. 10.1200/JCO.24.00826 PMC11687943 39121437

[45] Wang ES, Issa GC, Erba HP, Altman JK, Montesinos P, DeBotton S, et al. Ziftomenib in relapsed or refractory acute myeloid leukaemia (KOMET-001): a multicentre, open-label, multi-cohort, phase 1 trial. Lancet Oncol. 2024;25(10):1310–24. 39362248 10.1016/S1470-2045(24)00386-3

[46] Salzer E, Stutterheim J, Cuglievan B, Tomkinson BE, Leoni M, Van Tinteren H, et al. APAL2020K/ITCC-101: A Phase I Trial of the Menin Inhibitor Ziftomenib in Combination with Chemotherapy in Children with Relapsed/Refractory *KMT2A-*rearranged, *NUP98-*rearranged, *or NPM1-*mutant Acute Leukemias. Blood. 2024;144(Supplement 1):42653–3.

[47] Roberts AW. Therapeutic development and current uses of BCL-2 inhibition. Hematol Am Soc Hematol Educ Program. 2020;2020(1):1–9. 10.1182/hematology.2020000154 PMC7727569 33275682

[48] Cheung LC, Aya-Bonilla C, Cruickshank MN, Chiu SK, Kuek V, Anderson D, et al. Preclinical efficacy of Azacitidine and venetoclax for infant KMT2A-rearranged acute lymphoblastic leukemia reveals a new therapeutic strategy. Leukemia. 2023;37(1):61–71. 36380143 10.1038/s41375-022-01746-3 PMC9883157

[49] Gibson A, Trabal A, McCall D, Khazal S, Toepfer L, Bell DH, et al. Venetoclax for children and adolescents with acute lymphoblastic leukemia and lymphoblastic lymphoma. Cancers (Basel). 2021. 10.3390/cancers14010150 PMC8750927 35008312

[50] Place AE, Karol SE, Forlenza CJ, Cooper TM, Fraser C, Cario G, et al. Venetoclax combined with chemotherapy in pediatric and adolescent/young adult patients with relapsed/refractory acute lymphoblastic leukemia. Pediatr Blood Cancer. 2025. 10.1002/pbc.31630. 40062648 10.1002/pbc.31630

[51] Chatzikalil E, Roka K, Diamantopoulos PT, Rigatou E, Avgerinou G, Kattamis A, et al. Venetoclax combination treatment of acute myeloid leukemia in adolescents and young adult patients. J Clin Med. 2024. 10.3390/jcm13072046. 38610812 10.3390/jcm13072046 PMC11012941

[52] Bocuzzi V, Bridoux J, Pirotte M, Withofs N, Hustinx R, D’Huyvetter M, et al. CD38 as theranostic target in oncology. J Transl Med. 2024;22(1):998. 39501292 10.1186/s12967-024-05768-6 PMC11539646

[53] Sanchez L, Wang Y, Siegel DS, Wang ML. Daratumumab: a first-in-class CD38 monoclonal antibody for the treatment of multiple myeloma. J Hematol Oncol. 2016;9(1):51. 27363983 10.1186/s13045-016-0283-0 PMC4929758

[54] Darzalex [package insert]. Horsham, PA: Janssen Biotech, Inc; 2022.

[55] Bhatla T, Hogan LE, Teachey DT, Bautista F, Moppett J, Velasco Puyo P, et al. Daratumumab in pediatric relapsed/refractory acute lymphoblastic leukemia or lymphoblastic lymphoma: the DELPHINUS study. Blood. 2024;144(21):2237–47. 39158071 10.1182/blood.2024024493

